# Prognostic Implication of *KRAS* G12C Mutation in a Real-World *KRAS*-Mutated Stage IV NSCLC Cohort Treated With Immunotherapy in The Netherlands

**DOI:** 10.1016/j.jtocrr.2023.100543

**Published:** 2023-06-29

**Authors:** Anneloes L. Noordhof, Esther M. Swart, Ronald A.M. Damhuis, Lizza E.L. Hendriks, Peter W.A. Kunst, Mieke J. Aarts, Wouter H. van Geffen

**Affiliations:** a Department of Respiratory Medicine, Medical Center Leeuwarden, Leeuwarden, The Netherlands; b Department of Research & Development, Netherlands Comprehensive Cancer Organization, Utrecht, The Netherlands; c Department of Respiratory Medicine, GROW-School for Oncology and Reproduction, Maastricht University Medical Center, Maastricht, The Netherlands; d Department of Respiratory Medicine, Onze Lieve Vrouwe Gasthuis, Amsterdam, The Netherlands

**Keywords:** Non–small cell lung cancer, Kirsten rat sarcoma, *KRAS* G12C, (Chemo-)immunotherapy

## Abstract

**Introduction:**

With the approval of G12C inhibitors as the second line of treatment for *KRAS* G12C-mutated NSCLC, and the expanding research regarding targeting *KRAS*, it is key to understand the prognostic implication of *KRAS* G12C in the current first line of treatment. We compared overall survival (OS) of patients with stage IV *KRAS* G12C-mutated NSCLC to those with a *KRAS* non-G12C mutation in a first-line setting of (chemo)immunotherapy.

**Methods:**

This nationwide population-based study used real-world data from The Netherlands Cancer Registry. We selected patients with stage IV *KRAS*-mutated lung adenocarcinoma diagnosed in 2019 to 2020 who received first-line (chemo-)immunotherapy. Primary outcome was OS.

**Results:**

From 28,120 registered patients with lung cancer, 1185 were selected with a *KRAS* mutation, of which 494 had a *KRAS* G12C mutation. Median OS was 15.5 months (95% confidence interval [CI]: 13.6–18.4) for *KRAS* G12C versus 14.0 months (95% CI:11.2–15.7) for *KRAS* non-G12C (*p* = 0.67). In multivariable analysis, *KRAS* subtype was not associated with OS (hazard ratio = 0.95, 95% CI: 0.82–1.10). For the subgroup with programmed death-ligand 1 at 0% to 49% who received chemoimmunotherapy, median OS was 13.3 months (95% CI: 10.5–15.2) for G12C and 9.8 months (95% CI: 8.6–11.3) for non-G12C (*p* = 0.48). For the subgroup with programmed death-ligand 1 more than or equal to 50% who received monoimmunotherapy, the median OS was 22.0 months (95% CI: 18.4–27.3) for G12C and 18.9 months (95% CI: 14.9–25.2) for non-G12C (*p* = 0.36).

**Conclusions:**

There was no influence of *KRAS* subtype (G12C versus non-G12C) on OS in patients with *KRAS*-mutated stage IV NSCLC treated with first-line (chemo)immunotherapy.

## Introduction

*KRAS* is the most common driver mutation in patients with NSCLC, occurring in 30% to 40% of all NSCLC adenocarcinomas in western populations.^1^, ^2^, ^3^, ^4^ Through downstream signaling, mutations in *KRAS* can lead to uncontrolled cell growth and proliferation.^1^^,^^5^^,^^6^

Of all *KRAS* mutations, the *KRAS* G12C mutation is found in approximately 40% to 45% of all patients with *KRAS*-mutated NSCLC.^2^^,^^7^, ^8^, ^9^, ^10^

In the past, *KRAS* was considered to be “undruggable,” as several attempts to target this driver mutation were unsuccessful.^6^^,^^11^, ^12^, ^13^, ^14^ Recently, small molecules such as sotorasib and adagrasib were successfully able to keep the *KRAS* G12C molecule in its inactive state preventing downstream signaling.^15^, ^16^, ^17^ In clinical phase 2 studies, sotorasib and adagrasib were found to have an overall response rate (ORR) of 41% and 42.9% and a median progression-free survival (PFS) of 6.3 and 6.5 months, respectively, in patients with progressive disease after at least one previous line of systemic therapy.^18^^,^^19^ In the subsequent CodeBreaK 200 phase 3 trial, sotorasib had a superior median PFS than docetaxel (5.6 mo for sotorasib versus 4.5 mo for docetaxel, hazard ratio [HR] = 0.66, *p* = 0.0017). The PFS rate at 12 months was 24.8% for sotorasib and 10.1% for docetaxel.^20^^,^^21^ Covalent inhibitors of *KRAS* G12C have now become available as a second line of therapy for *KRAS* G12C-mutated NSCLC.^22^^,^^23^

Considering the new benchmark for *KRAS* G12C in this second line and the expanding research efforts to move KRAS inhibitors to the first line, it is key to understand the prognostic implication of *KRAS* G12C in the current first-line setting of treatment with immune checkpoint inhibitors or combination chemoimmunotherapy according to the tumor programmed death-ligand 1 (PD-L1) status. The prognostic role of *KRAS* G12C is currently unknown, as large real-world series regarding outcomes of patients with *KRAS* G12C-mutated NSCLC treated with (chemo)immunotherapy in a first-line setting are lacking. Only for unspecified *KRAS* mutations it has been found that there was no prognostic value of the *KRAS* mutational status in patients with stage IV NSCLC treated with first-line pembrolizumab.^24^ Nevertheless, regarding the prognostic role of *KRAS* G12C, current available literature is inconclusive as these studies were performed in small, selected populations.^9^^,^^25^, ^26^, ^27^, ^28^, ^29^, ^30^, ^31^, ^32^, ^33^, ^34^

To assess this real-world prognostic implication of *KRAS* G12C in relation with other *KRAS* subtypes, a large cohort study is needed, preferably in a real-world population. Using a population-based study in a nationwide cohort with real-world data, we aim to describe overall survival (OS) and to evaluate the prognostic implication of *KRAS* G12C for treatment with first-line monoimmunotherapy and combination immuno-chemotherapy in patients with lung adenocarcinoma compared with those with a *KRAS* non-G12C mutation.

## Materials and Methods

### Study Design

In this nationwide population-based study, real-world data from The Netherlands Cancer Registry (NCR) were used. The NCR is a population-based cancer registry that contains data of all individuals newly diagnosed with cancer in The Netherlands.^35^^,^^36^ Trained registration employees routinely collect data on patient and tumor characteristics (e.g., TNM classification of malignant tumors edition 8, WHO performance score [PS]), diagnostics, and treatments prescribed in the first line. For NSCLC, PD-L1 tumor proportion score and driver mutations are also reported. From patients diagnosed from 2019, information on *KRAS* subtype (G12C versus non-G12C) was collected. This retrospective, noninterventional study did not require approval from an accredited medical ethics committee or the Central Committee on Research involving Human Subjects. The study, however, has been reviewed and approved by the NCR’s Supervisory Committee and the multidisciplinary scientific committee regarding lung cancer (application number K22.135).

### Study Population

From the NCR, we selected all adult patients (aged ≥18 y) newly diagnosed with stage IV (as defined by clinical TNM IVA–B, TNM classification of malignant tumors eighth edition) *KRAS*-mutated NSCLC between January 1, 2019, and December 31, 2020. In addition, we restricted the cohort to patients diagnosed with the adenocarcinoma subtype and patients who received first-line treatment with immunotherapy with or without chemotherapy within 90 days of stage IV diagnosis. We excluded patients who tested also positive for other molecular driver alterations. Patients with an unknown PD-L1 expression level were also excluded.

### Statistical Analyses

Summary statistics were used to describe patient demographics, clinical characteristics, and tumor characteristics. OS was calculated from the start date of first-line systemic treatment to date of death from any cause. Patients without evidence of death were censored on February 1, 2022. The primary end point of this study was OS in which patients with *KRAS* G12C were compared with patients with *KRAS* non-G12C. OS was presented as 1-year and 2-year OS, and median survival time with corresponding 95% confidence intervals (CIs) using Kaplan-Meier curves. Log-rank tests were used to test for significant differences between the subgroups. Cox proportional hazards regression was used to assess HRs and 95% CIs in multivariable analysis including age, sex, WHO PS, number of organs with metastases, PD-L1 expression, year of diagnosis, and *KRAS* status. PD-L1 expression was included in potentially biological relevant cutoffs; therefore, next to the common cutoff values of 0% and 1% to 49%, we applied 50% to 89% and more than or equal to 90%. *KRAS* status was maintained in the final model, irrespective of prognostic significance. Other independent prognostic factors were determined using the backward selection method, where a *p* value of less than 0.05 was considered significant. Treatment type was not included in the multivariable model owing to collinearity with PD-L1. All analyses were performed using SAS, version 9.4 (SAS Institute, Cary, NC).

## Results

**Figure 1:**
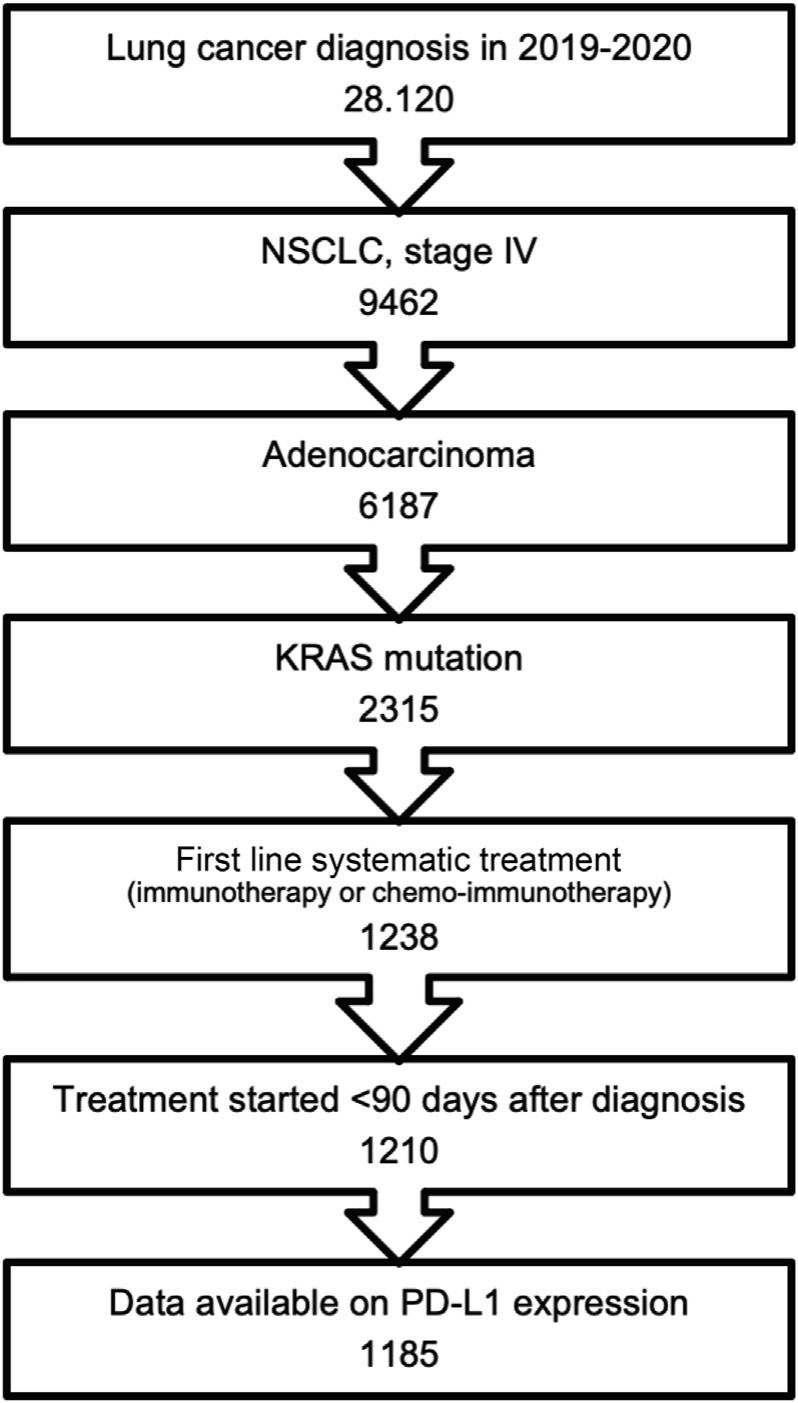
Flowchart patient selection. PD-L1, programmed death-ligand 1.

The NCR included a total of 28,120 patients with lung cancer in the study period, of whom a total of 1185 patients were selected (Fig. 1) with stage IV *KRAS*-positive adenocarcinoma of the lung, being treated with first-line immunotherapy or combination chemoimmunotherapy. Patient characteristics are outlined in Table 1. Of these patients, 494 had *KRAS* G12C-mutated NSCLC (41.7%), and the other 691 (58.3%) had non-G12C *KRAS*-mutated NSCLC. Of all included patients, 55.5% were female. Most of the patients had a WHO PS of 0 or 1 (81.2%).

**Table 1:** Patient Characteristics

	Overall (N = 1185),	*KRAS* G12C (n = 494),	*KRAS* Non-G12c (n = 691),
Characteristic	n (%)	n (%)	n (%)
Age			
18–59	317 (26.8)	128 (25.9)	189 (27.4)
60–69	477 (40.3)	206 (41.7)	271 (39.2)
≥70	391 (33.0)	160 (32.4)	231 (33.4)
Sex			
Men	527 (44.5)	216 (43.7)	311 (45.0)
Women	658 (55.5)	278 (56.3)	380 (55.0)
WHO performance score			
0	444 (37.5)	195 (39.5)	249 (36.0)
1	518 (43.7)	207 (41.9)	311 (45.0)
≥2	99 (8.4)	42 (8.5)	57 (8.2)
Unknown	124 (10.5)	50 (10.1)	74 (10.7)
Number of metastatic organs			
1	501 (42.3)	202 (40.9)	299 (43.3)
2	341 (28.8)	145 (29.4)	196 (28.4)
≥3	343 (28.9)	147 (29.8)	196 (28.4)
PD-L1			
0%	317 (26.8)	132 (26.7)	185 (26.8)
1%–49%	292 (24.6)	121 (24.5)	171 (24.7)
50%–89%	378 (31.9)	158 (32.0)	220 (31.8)
≥90%	198 (16.7)	83 (16.8)	115 (16.6)
Year			
2019	572 (48.3)	224 (45.3)	348 (50.4)
2020	613 (51.7)	270 (54.7)	343 (49.6)
Treatment			
Chemoimmunotherapy	735 (62.0)	302 (61.1)	433 (62.7)
Monoimmunotherapy	450 (38.0)	192 (38.9)	258 (37.3)

PD-L1, programmed death-ligand 1.

The percentage of patients with *KRAS* G12C was slightly lower in 2019 compared with 2020. Distinction between the *KRAS* subtypes was introduced at the NCR in 2019, and some miscoding may have taken place. Nevertheless, similar results were obtained in sensitivity analyses stratifying for period of diagnosis.

Of all patients, 62% were treated with chemoimmunotherapy and 38% with monoimmunotherapy. In addition, of all patients with a PD-L1 expression more than or equal to 50%, 77% received monoimmunotherapy and the other 23% combination chemoimmunotherapy. From all patients with a tumor PD-L1 expression less than 50%, 99% received combination chemoimmunotherapy (Supplementary Table A.1). Monoimmunotherapy comprised pembrolizumab in all the patients.

**Figure 2:**
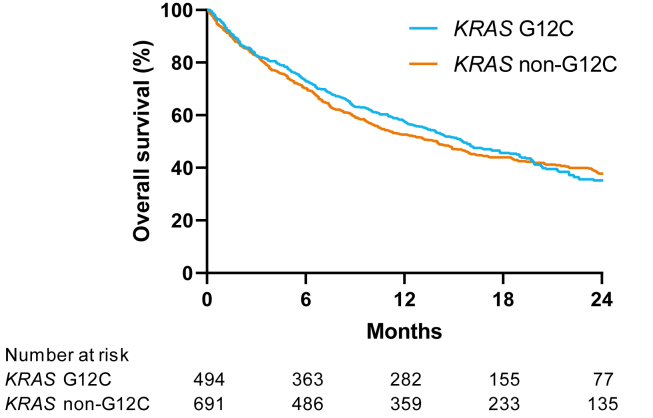
Kaplan-Meier overall survival *KRAS* non-G12C versus *KRAS* G12C.

The median OS, regardless of treatment type, of the patients with a *KRAS* G12C NSCLC was 15.5 months (95% CI: 13.6–18.4) versus 14.0 months (95% CI: 11.2–15.7) for *KRAS* non-G12C (*p* = 0.67; Fig. 2). In univariable analysis, sex was a significant prognostic factor for OS, as women had a superior survival compared with men (*p* < 0.0001). Associated with poorer survival were poorer WHO PS (*p* < 0.0001), higher number of metastatic organs (*p* < 0.0001), and lower PD-L1 expression (*p* < 0.0001). Type of treatment was also a significant prognostic factor for survival, as monoimmunotherapy was associated with a higher survival (*p* < 0.0001; Table 2).

**Table 2:** Univariable Analysis of Overall Survival

Variable	n	1-y, %	95% CI	2-y, %	95% CI	*p* Value
Age						
18–59	317	58	(53–64)	42	(36–48)	0.0044
60–69	477	57	(52–61)	37	(32–42)	
≥70	391	49	(44–54)	32	(27–37)	
Sex						
Men	527	49	(44–53)	31	(27–35)	<0.0001
Women	658	60	(56–63)	42	(37–46)	
WHO performance score						
0	444	65	(61–70)	46	(41–51)	<0.0001
1	518	50	(46–54)	32	(28–37)	
≥2	99	39	(30–49)	32	(23–42)	
Unknown	124	48	(39–56)	25	(16–34)	
Number of metastatic organs						
1	501	64	(59–68)	46	(41–51)	<0.0001
2	341	55	(50–60)	35	(29–41)	
≥3	343	41	(36–46)	25	(21–31)	
PD-L1						
0%	317	41	(36–46)	20	(16–26)	<0.0001
1%–49%	292	55	(49–61)	35	(29–41)	
50%–89%	378	59	(54–64)	45	(39–50)	
≥90%	198	67	(60–73)	50	(42–58)	
Year						
2019	572	57	(52–61)	38	(34–42)	0.3329
2020	613	53	(49–57)	-	(-)	
*KRAS*						
*KRAS* G12C	494	57	(53–62)	35	(30–40)	0.6683
*KRAS* non-G12C	691	53	(49–56)	38	(34–42)	
Treatment						
Chemoimmunotherapy	735	50	(47–54)	31	(28–35)	<0.0001
Monoimmunotherapy	450	62	(57–66)	46	(41–51)	

CI, confidence interval; PD-L1, programmed death-ligand 1.

Sex, WHO PS, number of metastatic organs, and PD-L1 status remained significant prognostic factors in multivariable analysis (Table 3). *KRAS* mutation status (i.e., G12C versus non-G12C) was in this multivariable analysis not significantly associated with OS (HR = 0.95, 95% CI: 0.82–1.10).

**Table 3:** Multivariable Analysis of Overall Survival

Variable	Hazard Ratio	95% CI
Age		
18–59	1	
60–69	1.17	(0.97–1.41)
≥70	1.39	(1.14–1.70)
Sex		
Men	1	
Women	0.83	(0.71–0.96)
WHO performance score		
0	1	
1	1.44	(1.22–1.71)
≥2	1.85	(1.41–2.44)
Unknown	1.75	(1.36–2.25)
Number of metastatic organs		
1	1	
2	1.30	(1.08–1.56)
≥3	2.02	(1.70–2.41)
PD-L1		
0%	1	
1%–49%	0.63	(0.51–0.76)
50%–89%	0.53	(0.44–0.64)
≥90%	0.38	(0.30–0.49)
Year		
2019	-	
2020		
*KRAS*		
*KRAS* non-G12C	1	
*KRAS* G12C	0.95	(0.82–1.10)

CI, confidence interval; PD-L1, programmed death-ligand 1.

For the subgroup with PD-L1 at 0% to 49% who received combination chemoimmunotherapy, median OS was 13.3 months (95% CI: 10.5–15.2) for G12C and 9.8 months (95% CI: 8.6–11.3) for non-G12C (*p* = 0.48; Supplementary Fig. A.1*A*). No differences were observed in the subgroup with a tumor PD-L1 expression of 0% (median OS for G12C at 10.0 mo [95% CI: 6.6–13.3] versus median OS for non-G12C at 8.8 mo [95% CI: 6.8–9.8]; *p* = 0.80; Supplementary Fig. A.1*B*) and in the subgroup with PD-L1 at 1% to 49% (median OS for G12C at 16.0 mo [95% CI: 13.1–21.3] versus median OS for non-G12C at 12.2 mo [95% CI: 10.0–19.4], *p* = 0.43; Supplementary Fig. A.1*C*).

**Figure 3:**
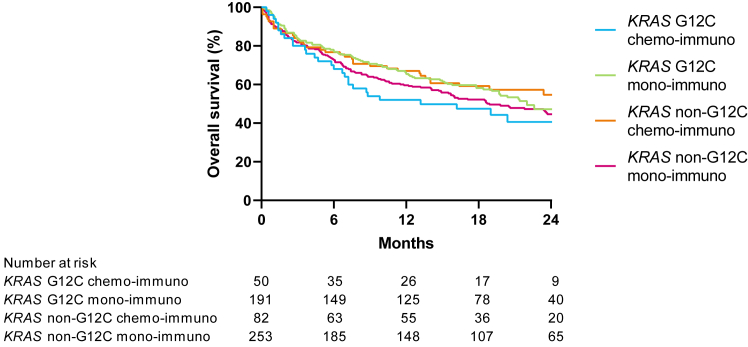
Kaplan-Meier subgroup with PD-L1 more than or equal to 50% by treatment for *KRAS* non-G12C versus *KRAS* G12C. PD-L1, programmed death-ligand 1.

Different treatment strategies were used in patients with a PD-L1 expression more than or equal to 50% (Supplementary Table A.1). The median OS of patients with a PD-L1 expression more than or equal to 50% who were treated with monoimmunotherapy was 22.0 months (95% CI: 18.4–27.3) for G12C and 18.9 months (95% CI: 14.9–25.2) for non-G12C (*p* = 0.36; Supplementary Fig. A.2). Patients with a PD-L1 expression more than or equal to 50% who received combination chemoimmunotherapy had a median OS of 13.2 months (95% CI: 6.9–not evaluable) for those with *KRAS* G12C-mutated NSCLC and 30.5 months (95% CI:14.0–not evaluable) for those with *KRAS* non–G12C-mutated NSCLC (Fig. 3).

We observed no significant difference in OS between the two treatment strategies for patients with PD-L1 expression more than or equal to 50% in regard to treatment type (monoimmunotherapy or combination chemoimmunotherapy) and *KRAS* mutational status (*KRAS* G12C or *KRAS* non-G12C) (*p* = 0.32; Fig. 3).

## Discussion

In this nationwide population-based study using real-world data, the OS of patients with stage IV NSCLC with a *KRAS* G12C versus a non-G12C mutation was similar, when treated with first-line monoimmunotherapy or combination chemoimmunotherapy. Unspecified *KRAS* mutational status was already known to have no prognostic impact on OS in patients with stage IV lung adenocarcinoma.^24^^,^^34^ This study established that the G12C subtype neither influences survival.

In the KEYNOTE-042 study, patients with *KRAS* G12C-positive NSCLC had similar ORR, PFS, and OS compared with the overall cohort of patients with unspecified *KRAS*-mutated NSCLC while being treated with pembrolizumab monotherapy.^37^^,^^38^ In the KEYNOTE-189, it was found that patients with *KRAS* G12C-positive NSCLC had similar ORR compared with all patients with unspecified *KRAS*-mutated NSCLC after treatment with pembrolizumab plus chemotherapy.^39^^,^^40^ Nevertheless, these were post hoc analyses with a relatively small number of patients.

Justeau et al.^34^ found in a smaller and more selected *KRAS*-positive stage IV NSCLC cohort (with only PD-L1 ≥50%, treated with first-line immunotherapy) an OS of 18.4 months versus 20.6 months for *KRAS* G12C (n = 86) versus non-G12C (n = 141), respectively. These results are similar to the OS reported in the current study. Other previous studies lacked conclusive evidence regarding the real-world prognostic value of *KRAS* G12C, mainly owing to limited sample sizes, and these studies were rarely performed in a first-line setting.^9^^,^^25^^,^^27^, ^28^, ^29^, ^30^, ^31^

In our cohort, 55.5% of all patients with a *KRAS* mutation were female. This contrasts with two large cohorts from France and Germany^10^^,^^41^ but corresponds with other studies.^2^^,^^8^^,^^9^^,^^42^ In our study, sex was a significant prognostic factor for OS, as women had a better survival than men which was not explained by differences in patient and tumor characteristics (*p* < 0.0001). This was not found in other larger *KRAS* cohorts, but in these cohorts, patients with stages I to IV NSCLC with different treatment modalities were included,^42^^,^^43^ and our study included only those with stage IV treated with systemic therapy. This could mean that there are sex-specific influences in patients with stage IV NSCLC with a *KRAS* mutation.

Strengths of this study are that it is a nationwide population-based study using real-world data and, to our knowledge, the largest cohort to evaluate the prognostic implication of *KRAS* G12C in the setting of first-line (chemo)immunotherapy.

As this study used NCR data, no data were available regarding PFS, compliance to treatment, comorbidity, or cause of death. In addition, data containing co-mutations were not included in the data set. *KRAS* can have co-occurring mutations in *TP53*, *STK11*, and *KEAP1*, with a prevalence of 39% to 48%, 12% to 30%, and 8% to 27%, respectively.^9^^,^^43^, ^44^, ^45^, ^46^ In a large study, the frequencies of *TP53*, *STK11*, and *KEAP1* were similar in *KRAS* G12C and non–G12C-mutated lung cancer.^9^ In literature, patients with a *KRAS* G12C and co-occurring *STK11* mutation had a significant shorter time to next treatment and shorter OS with immunotherapy or combination chemoimmunotherapy than patients without the co-occurring *STK11* mutation.^47^ Patients with *KRAS* and co-occurring *KEAP1* mutation had a shorter OS with platinum-based chemotherapy and immunotherapy.^44^^,^^48^ In the sotorasib phase 2 study, patients with *KRAS* G12C-mutated NSCLC who had *STK11* wild type but had a *KEAP1* alteration seemed to have the lowest response rate, although this was not sufficiently powered for statistical analysis.^18^ A similar effect was found with adagrasib, suggesting that this *KEAP1* co-occurring mutation has an impact on treatment response.^19^

In this real-world study, 77% of the patients with a PD-L1 expression of more than or equal to 50% were treated with monoimmunotherapy. The other 23% was treated with combination chemoimmunotherapy and could possibly reflect a population with a higher tumor burden and more rapid progressive disease. To prevent bias due to this, separate survival analysis was performed in the subgroup with PD-L1 more than or equal to 50%, for those treated with monoimmunotherapy and those with combination chemoimmunotherapy.

Furthermore, it is important to notice that this study was performed in a predominantly white population. In western populations, *KRAS* mutations are more frequently reported than in populations from Asian descent.^49^ The G12C mutation is observed more often in white and black women than in white and black men, but more often in Asian men than in Asian women.^50^

A subject for future research is whether co-occurring alterations with *KRAS* G12C have a predictive role on survival. Furthermore, one could hypothesize if there is a subgroup that could possibly benefit from G12C inhibitors instead of (chemo-)immunotherapy or in combination strategies in the first line of therapy. Further research should therefore focus on these topics, now that G12C inhibitors have become available in the therapeutic landscape. Although we did not find survival differences between *KRAS* non-G12C and *KRAS* G12C within the 2-year follow-up period, survival differences might emerge over time, because a considerable number of patients were still alive at the end of the follow-up. The relatively lower OS observed in the subgroup with PD-L1 at 0% to 49% who received combination chemoimmunotherapy highlights the need for further research on how to optimize treatment strategies for this subgroup.

In conclusion, we established that the *KRAS* G12C mutational status was not associated with OS in the first-line setting of patients with stage IV *KRAS*-mutated NSCLC treated with (chemo-)immunotherapy. For now, PD-L1 status is the most important prognostic biomarker currently available for *KRAS*-mutant NSCLC. Furthermore, no survival differences were found for the different treatment strategies (monoimmunotherapy versus combination chemoimmunotherapy) used in the subgroup with PD-L1 more than or equal to 50%.

## CRediT Authorship Contribution Statement

**Anneloes L. Noordhof:** Conceptualization, Writing—original draft, Visualization, Project administration.

**Esther M. Swart:** Conceptualization, Methodology, Formal analysis, Writing—review and editing.

**Ronald A. M. Damhuis:** Methodology, Writing—review and editing.

**Lizza E. L. Hendriks:** Writing—review and editing.

**Peter W. A. Kunst:** Writing—review and editing.

**Mieke J. Aarts:** Conceptualization, Methodology, Formal analysis, Writing—review and editing, Supervision.

**Wouter H. van Geffen:** Conceptualization, Methodology, Writing—review and editing, Supervision.

## Supplementary Data

Figure A 1A

Figure A 1B

Figure A 1C

Figure A 2

Table A1
