# Supplementary figures and images for: Prognostic Implication of KRAS G12C Mutation in a Real-World KRAS-Mutated Stage IV NSCLC Cohort Treated With Immunotherapy in The Netherlands
Source: JTO Clin Res Rep. 2023 Jun 29;4(9):100543. doi: 10.1016/j.jtocrr.2023.100543 (PMC10477684; doi:10.1016/j.jtocrr.2023.100543)

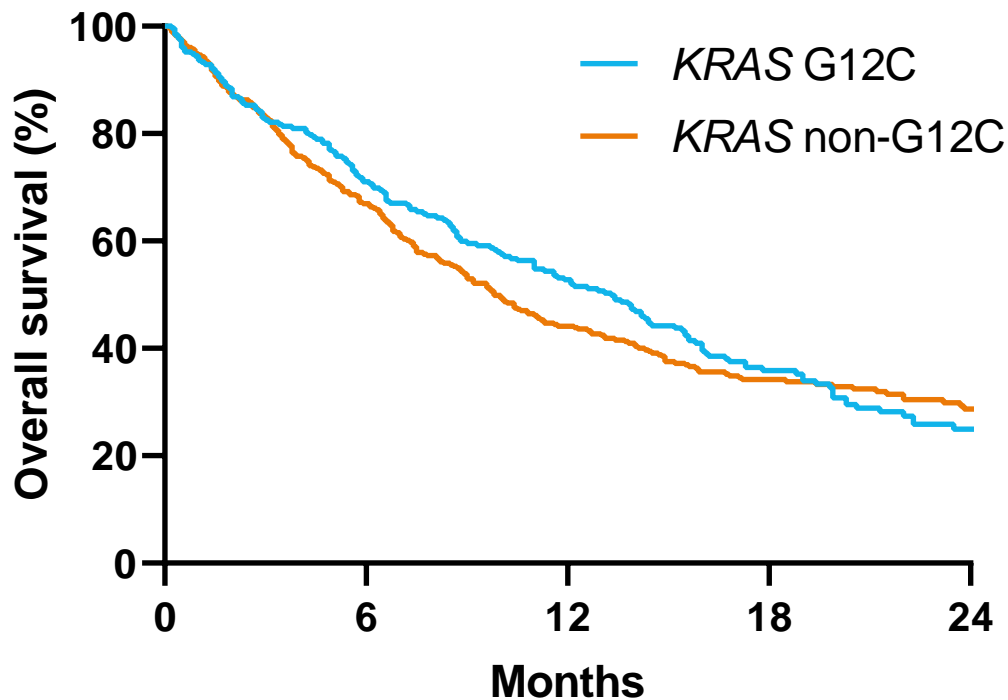

Number at risk

|                      |     |     |     |    |    |
|----------------------|-----|-----|-----|----|----|
| <i>KRAS</i> G12C     | 252 | 179 | 131 | 60 | 28 |
| <i>KRAS</i> non-G12C | 351 | 235 | 154 | 89 | 49 |

Supplement: Figure A 1A [file mmc1.pdf]

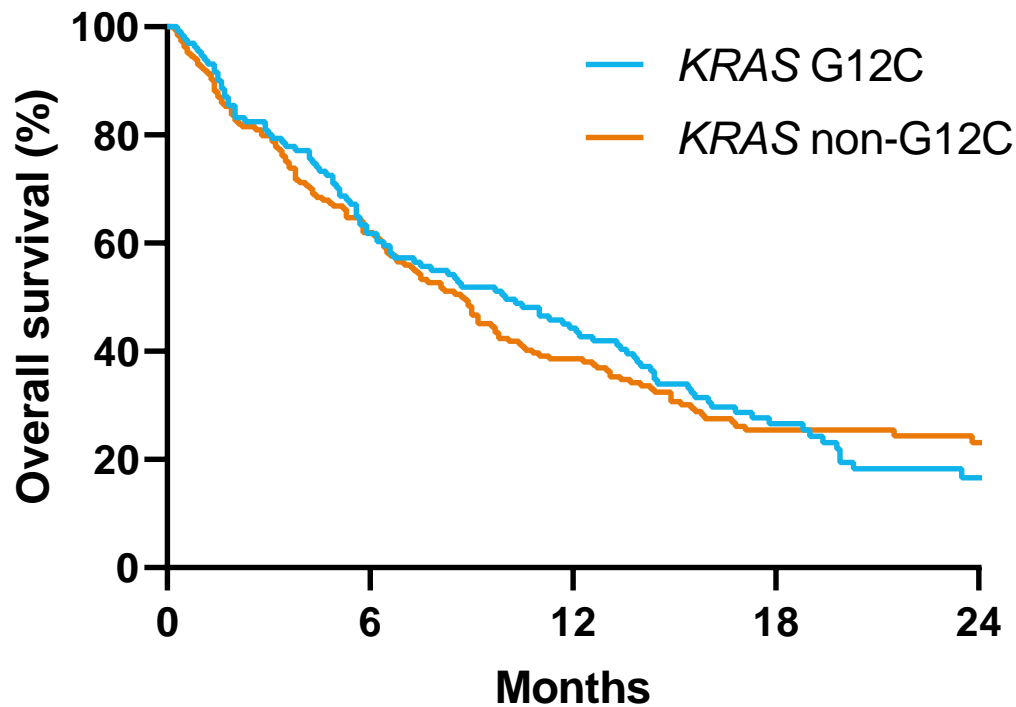

Number at risk

|                      |     |     |    |    |    |
|----------------------|-----|-----|----|----|----|
| <i>KRAS</i> G12C     | 131 | 81  | 58 | 23 | 10 |
| <i>KRAS</i> non-G12C | 184 | 114 | 71 | 33 | 19 |

Supplement: Figure A 1B [file mmc2.pdf]

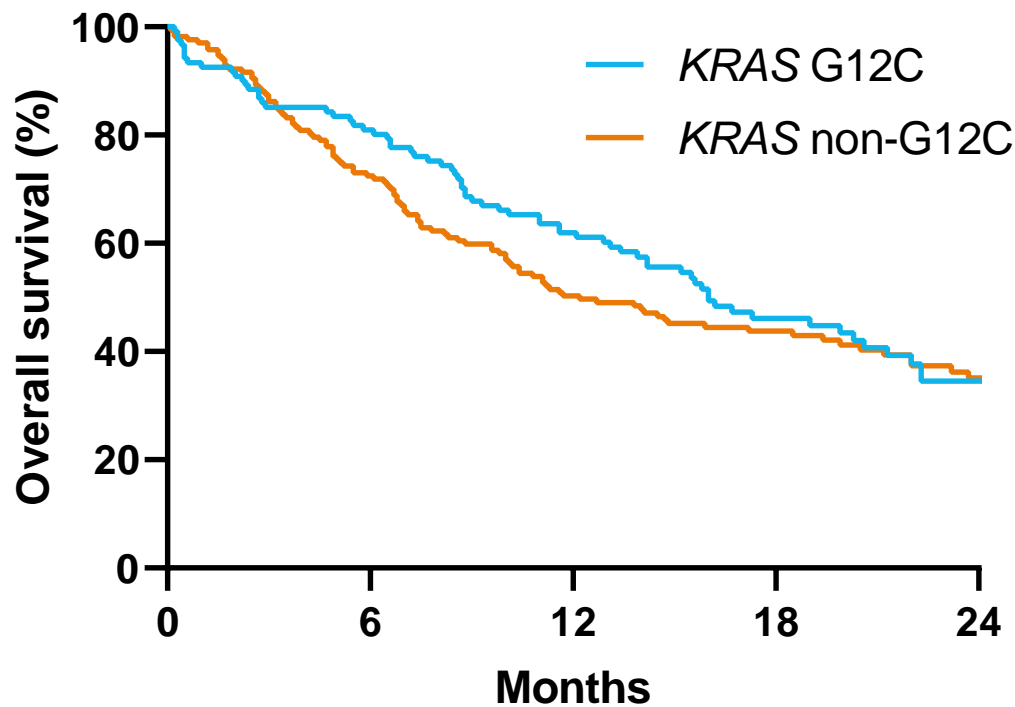

Number at risk

|                      |     |     |    |    |    |
|----------------------|-----|-----|----|----|----|
| <i>KRAS</i> G12C     | 121 | 98  | 73 | 37 | 18 |
| <i>KRAS</i> non-G12C | 167 | 121 | 83 | 56 | 30 |

Supplement: Figure A 1C [file mmc3.pdf]

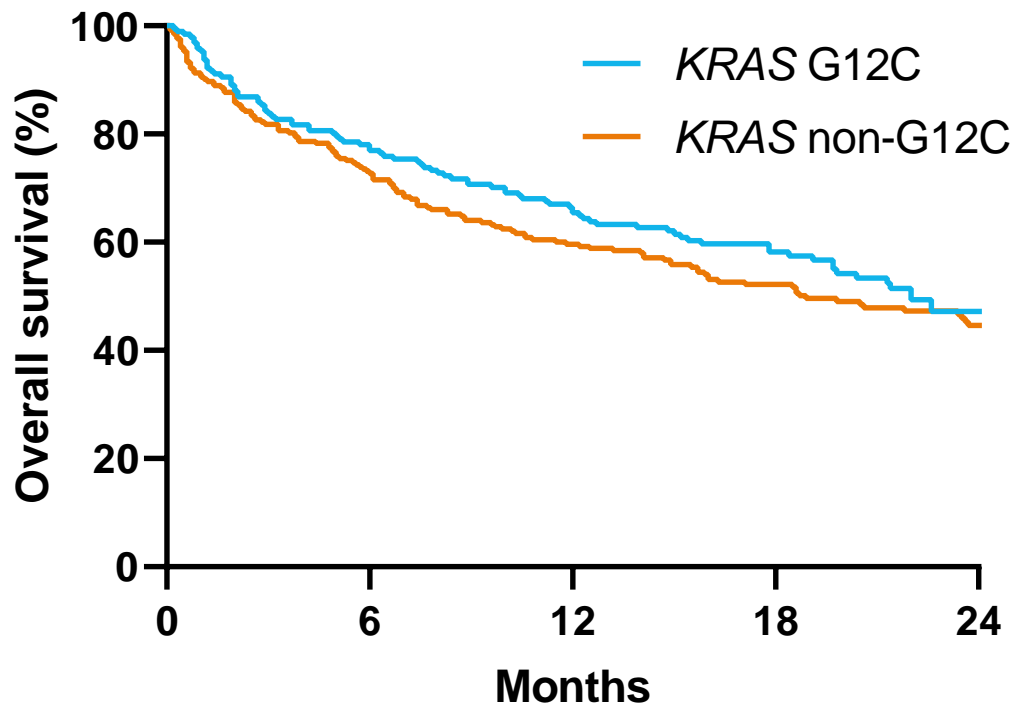

Number at risk

|                      |     |     |     |     |    |
|----------------------|-----|-----|-----|-----|----|
| <i>KRAS</i> G12C     | 191 | 149 | 125 | 78  | 40 |
| <i>KRAS</i> non-G12C | 253 | 185 | 148 | 107 | 65 |

Supplement: Figure A 2 [file mmc4.pdf]
