# Supplementary material for: Prognostic Implication of KRAS G12C Mutation in a Real-World KRAS-Mutated Stage IV NSCLC Cohort Treated With Immunotherapy in The Netherlands
Source: JTO Clin Res Rep. 2023 Jun 29;4(9):100543. doi: 10.1016/j.jtocrr.2023.100543 (PMC10477684; doi:10.1016/j.jtocrr.2023.100543)
Supplement: Table A1 [file mmc5.docx]

Table A.1. Treatment per PD-L1 expression group

|  | PD-L1 0-49%  n (%) | PD-L1 ≥50%  n (%) |
| --- | --- | --- |
| Mono-immunotherapy  Pembrolizumab | 6 (1%) | 444 (77%) |
| Chemo-immunotherapy  Carboplatin-pemetrexed-pembrolizumab | 603 (99%)  461 (75.7%) | 132 (23%)  96 (16.7%) |
| Cisplatin-pemetrexed-pembrolizumab | 83 (13.6%) | 20 (3.5%) |
| Other | 59 (9.7%) | 16 (2.8%) |

Abbreviations: PD-L1, programmed death ligand 1.
